# The evolution of transmission mode

**DOI:** 10.1098/rstb.2016.0083

**Published:** 2017-03-13

**Authors:** Janis Antonovics, Anthony J. Wilson, Mark R. Forbes, Heidi C. Hauffe, Eva R. Kallio, Helen C. Leggett, Ben Longdon, Beth Okamura, Steven M. Sait, Joanne P. Webster

**Affiliations:** 1 Department of Biology, University of Virginia, Charlottesville, VA 22904, USA; 2 Integrative Entomology group, Vector-borne Viral Diseases programme, The Pirbright Institute, Pirbright GU24 0NF, UK; 3 Department of Biology, Carleton University, 1125 Colonel By Drive, Ottawa, Ontario, Canada K1S 5B7; 4 Department of Biodiversity and Molecular Ecology, Research and Innovation Centre, Fondazione Edmund Mach, Via E. Mach 1, 38010 S. Michele all'Adige, Trentino, Italy; 5 Department of Biological and Environmental Science, University of Jyvaskyla, PO Box 35, 40014 Jyvaskyla, Finland; 6 Department of Ecology, University of Oulu, PO Box 3000, 90014 Oulu, Finland; 7 Department of Genetics, University of Cambridge, Cambridge CB2 3EH, UK; 8 Centre for Ecology and Conservation, University of Exeter, Penryn Campus, Cornwall TR10 9FE, UK; 9 Department of Life Sciences, Natural History Museum, Cromwell Road, London SW5 7BD, UK; 10 School of Biology, University of Leeds, Leeds LS2 9JT, UK; 11 Department of Pathology and Pathogen Biology, Centre for Emerging, Endemic and Exotic Diseases, Royal Veterinary College, University of London, London AL9 7TA, UK

**Keywords:** infectious disease, host shifts, complex life cycles, spill-over

## Abstract

This article reviews research on the evolutionary mechanisms leading to different transmission modes. Such modes are often under genetic control of the host or the pathogen, and often in conflict with each other via trade-offs. Transmission modes may vary among pathogen strains and among host populations. Evolutionary changes in transmission mode have been inferred through experimental and phylogenetic studies, including changes in transmission associated with host shifts and with evolution of the unusually complex life cycles of many parasites. Understanding the forces that determine the evolution of particular transmission modes presents a fascinating medley of problems for which there is a lack of good data and often a lack of conceptual understanding or appropriate methodologies. Our best information comes from studies that have been focused on the vertical versus horizontal transmission dichotomy. With other kinds of transitions, theoretical approaches combining epidemiology and population genetics are providing guidelines for determining when and how rapidly new transmission modes may evolve, but these are still in need of empirical investigation and application to particular cases. Obtaining such knowledge is a matter of urgency in relation to extant disease threats.

This article is part of the themed issue ‘Opening the black box: re-examining the ecology and evolution of parasite transmission’.

## 1. Introduction

Transmission is central to disease biology and epidemiology, and the transmission modes of pathogens and parasites are complex and diverse. However, there has been limited attention given to how transmission mode evolves, especially in comparison with other evolutionary outcomes of disease interactions such as co-evolution during the infection process [1], the evolution of host-range [2,3], or the evolution of virulence ([4,5], see also [6]). This review examines major issues and findings relating to the evolution of transmission mode. We focus on the evolution of transmission as a trait in its own right, and only tangentially consider how different transmission modes once established have evolutionary consequences for disease expression and virulence as these have been the subject of other reviews [4,5,7–11].

Our review broadly addresses the following questions:

— What are the types of transmission and how can be they studied? We address some awkward semantic and methodological problems unique to studying transmission modes and routes.
— How does transmission mode evolve? At a micro-evolutionary scale, we examine the evidence for genetic variation in transmission mode and the nature of the trade-offs involved, including evidence from selection experiments.
— What are the predictions of population genetic models about directions of evolution in transmission mode? When will there be stable genetic variation for transmission mode and when will mixed modes be favoured?
— What directions has the evolution of transmission mode taken in the past? We review phylogenetic and comparative studies on changes in transmission mode, asking if there are preferred evolutionary pathways, and what forces might lead to them.
— Do changes in transmission mode accompany host shifts or emergence of new diseases? We examine the evidence, and emphasize the importance of understanding this process in dealing with newly emerging diseases.
— Throughout, we emphasize that the evolution of ‘transmission mode’ is determined by the genotypes of both the pathogen and the host, and is a co-evolutionary process, not just an evolved property of the pathogen.

## 2. Transmission modes and routes

The transmission of parasites and pathogens is often referred to in the literature and public health information sites as having various ‘modes’ and ‘routes’; however, these two terms are used interchangeably, which confuses two concepts important for evaluating the process whereby transmission evolves. In common usage, a ‘mode’ of transport (e.g. train, bus, car and bicycle) is easily distinguishable from a ‘route’ taken to get to a destination (e.g. via which city, or via which specific international departure and arrival point). Similarly, in reference to transmission, ‘mode’ should refer to the method that a pathogen uses to get from starting point to destination, whereas the ‘route’ is the path taken using the chosen mode and includes a starting point (site of pathogen presentation, or portal of exit), a specific pathway used, and a destination (where the pathogen enters). This distinction is important because the mode defines certain epidemiological characteristics of the pathogen and the disease, and hence expectations for its possible evolution (for example, sexual versus non-sexual transmission [12]). The routes for one mode may be several, or many, and dictate specifically *how* the pathogen will leave one body and infect another, e.g. faecal–oral, hand–oral, fomite–lung, etc. (of course, knowing the route still does not tell you the mechanisms of infection, which are also incredibly varied!). Until we know both the mode and route, the transmission is not fully defined. For example, a pathogen transmitted by the lung-to-lung route may be droplet-borne or airborne, and a pathogen transmitted by the vertical mode may take the transplacental or vaginal-skin route. However, once we know both mode and route, the evolutionary trajectory may be hypothesized and control measures can be implemented. Knowledge of routes associated with a given mode might also indicate how restricted a particular pathogen might be in its transmission, which in turn may suggest more precise or wide-ranging methods of control. For example, airborne pathogens mainly spread from one respiratory tract to another, whereas vector-borne pathogens can be transmitted from vector to skin, from the vectors’ faeces to lung, or from a vector bite to the blood stream.

Modes can be subcategorized in various ways: one possibility is shown in table 1. The actual hierarchical order of the divisions and sub-divisions is debatable but these are the commonly used dichotomies. Within the evolutionary literature on disease, the major distinction made among transmission modes is between vertical and horizontal transmission, with horizontal transmission commonly subdivided into sexual versus non-sexual. Most health and government organizations classify infectious diseases as being transmitted ‘directly’ (e.g. sexual, vertical, skin-to-skin contact) and ‘indirectly’ (e.g. airborne, vector-borne, vehicle-borne, water- and food-borne) [13–15]. As directly transmissible diseases are by definition spread by direct contact between individuals, this distinction may be more useful to warn medical workers that they may be at risk of infection by directly transmitted pathogens from their patients. Another distinction is sometimes made based on the form of the transmission function in relation to density of infected individuals, namely frequency-dependent versus density-dependent transmission [16,17].

**Table 1.** One of many possible classifications of transmission modes, to illustrate the use of the terms ‘mode’ and ‘route’, with the former term being used for the method of getting from point to destination, and the latter for the path taken, which includes the points of exit and entry. The table is not intended to be definitive or comprehensive; thus, for example, vector transmission could be further subdivided into passive or biological, and the latter into multiplicative or non-multiplicative/circulatory-only.

mode				route (examples)
vertical				cytoplasmic, transplacental, during vaginal birth or breast feeding
horizontal	sexual			mainly genital–genital, but also oro-genital, flower to flower
	non-sexual	direct contact		skin-to-skin: kissing, biting, touching
		airborne		respiratory tract–respiratory tract
		indirect	environmental	contaminated food–oral, infected water–oral, faecal–oral, water–skin as in helminths
			fomites	clothing–skin, needle–blood, doorknob–hand
			vector-borne	cutaneous penetration; vector fecal deposition, vector identity

Surprisingly, the terms ‘movement’ and ‘dispersal’ appear rarely in the disease literature, and are generally considered to be processes that are components of transmission. Ecologists define dispersal as the movement of an individual from a source location to a new location [18,19], and ‘effective dispersal’ includes the added element of establishment and breeding in the new location. Therefore, transmission in the disease literature corresponds to the idea of effective dispersal in the ecological sphere.

## 3. Determining transmission modes and routes

Quantifying the contribution of different modes and routes to overall transmission of a pathogen is a major challenge, and the general lack of data on transmission for most pathogens is one of the greatest obstacles to studying its evolution. For example, as discussed below, understanding evolutionary pathways in transmission is more limited by reliable knowledge of the transmission mode than by the phylogenies of the pathogens involved [12]. Generally, three approaches have been taken to establish and measure transmission mode: genetic studies involving markers, observation of contact processes and experimental studies. The presence of congruent host and pathogen phylogenies has also been used to infer that in the past pathogen transmission has been predominantly vertical [10,20]. However, this interpretation has been questioned because congruent phylogenies may also result from the greater likelihood of host shifts between related taxa by horizontal transmission [21–23]. Moreover, a high level of observed vertical transmission does not preclude a horizontal transmission route as the latter may be essential to maintain a high disease prevalence, in turn resulting in high effective vertical transmission [24].

Most infectious diseases have the potential to be transmitted by multiple modes, so a major issue is determining which modes are the most important in a particular host–pathogen system. Even modes that appear ‘incidental’ or unimportant, may, if they have a genetic basis, be the target of selection in novel circumstances. A classic example is the protozoan *Toxoplasma gondii*. While the one definitive host, a species of Felidae, sheds oocysts in the stool, these can infect most warm-blooded organisms when they consume contaminated vegetation or raw meat. Species such as sheep, humans, mice and rats can maintain infection through congenital or neonate transmission [25,26], and several cases of sexual transmission have also been documented in experimental studies [27–29]. Another example is Rift Valley fever virus (RVFV), which spreads among livestock through mosquito bites but can also be transmitted vertically [30]. RVFV is transmitted from domestic animals to humans mainly by direct contact with infected animals, consumption of raw milk, and in a few cases, through mosquito bites [31,32]. During inter-epidemic periods, RVFV may be maintained in some mosquito species by transovarial vertical transmission [33]. However, as with *Toxoplasma*, we know little about the strength of these different modes of transmission, and whether any of them involve unique genetic variants [34].

Epidemiological tracing using genetic markers might seem a particularly useful approach to studying transmission mode, but while markers can identify the source and target of a transmission event, they cannot *per se* pinpoint the transmission mode unless combined with other approaches. A classic example is the tracing of HIV infections to particular healthcare workers. However, only by assessing associated risk factors (e.g. sexual activity of the health care workers and patients) was it established that many of these HIV infections were likely to have been blood-borne rather than sexually transmitted [35]. Two more recent examples relate to the outbreaks of foot and mouth disease virus (FMDV) and bluetongue virus (BTV) in the UK. In the FMDV outbreak in 2001, subsequent sequencing of viral isolates confirmed most but not all inferences from contact tracing, including aerial spread, animals congregating at markets and direct transport of infected animals [36]; however, sequencing by itself could not have ‘established’ these routes. Genetic studies of BTV showed that the strain that caused the 2006 epidemic originated in sub-Saharan Africa, and was not vaccine-derived [37], but the pathway whereby it reached the UK could not be determined [38].

Genetic markers are perhaps most useful in determining transmission routes in multi-host systems. For example, microsatellite markers have been used to identify possible hosts of *Schistosoma japonicum* [39–41]. DNA sequencing analysis of mosquito blood meals was used to establish which bird species were potentially important for West Nile virus transmission to humans [42]. Transmission in multi-host systems is more extensively discussed in [3,43,44].

Studies of co-inheritance of genetic markers in parasites and both cytoplasmic and nuclear genetic markers in their hosts can also provide information on the degree to which transmission is vertical or horizontal [45]. Under perfect maternal transmission, there is complete linkage disequilibrium between host mtDNA and pathogen alleles, and degrees of departure from this can be used to back-infer the amount of horizontal transfer [46].

The comparison of patterns in pathogen phylogenetic distance is a related and promising approach to infer transmission mode. This approach can provide evidence for multiple transmission modes in a system, as different lineages may show different relationships. For example, if pathogen genetic distance between related hosts is less than expected by chance in some strains, it is likely that vertical transmission plays some role in their transmission mode, as has been demonstrated for feline immunodeficiency virus in lions (N. M. Fountain-Jones 2016, personal communication). Conversely, if there is a strong spatial pattern in pathogen genetic distance but little effect of host relatedness, it is possible that horizontal transmission is the dominant mode.

Experimental infections also provide estimates of the relative importance of different transmission routes. For example, in avian influenza, experimental infections have estimated persistence of virus in the environment, and thus the relative importance of airborne versus environmental (faecal–oral) routes [47–49]. Similarly, experimental studies on FMDV have used calves either directly exposed to other infected individuals or housed in buildings that had previously held infected individuals to study direct versus environmental transmission [26]. As another example, to determine whether vertical (congenital) transmission alone was sufficient to maintain transmission of *T. gondii* in brown rats, *Rattus norvegicus*, [26] rats were trapped from local farms and released into a large naturalistic outdoor enclosure in the absence of oocysts from the feline definitive host. Over the subsequent three years, the rat population expanded but the seroprevalence remained approximately constant, showing feline hosts were not essential to maintain transmission. Although entomopathogenic *Rickettsia* is generally assumed to be vertically transmitted, experimental studies [50] showed a phytophagous *Rickettsia* could be horizontally transmitted via the phloem; uninfected whiteflies (*Bimesa tabaci*) physically separated from infected whiteflies could acquire the infection by feeding on the same leaf.

Experimental studies exposing potential arthropod vectors to pathogens by allowing them to feed on infected hosts are relatively commonplace. The detection of the pathogens (often viral RNA) can be in the saliva or head of the insect [51,52] or in the whole insect [53,54]. However, most such studies implicitly assume that the demonstration of pathogen replication in a vector following artificial exposure to a pathogen is adequate to infer vector-borne transmission in the field. Unfortunately, studying actual transmission under field conditions is both expensive, time-consuming and rarely done [55].

Transmission mode can obviously be determined by many methods. Contact tracing and inferring transmission modes based on behaviours among contacts is a method commonly used in humans. Age specificity of infection, location of the pathogen, site of the lesions and the biology of the transmission stages are all pointers to the transmission mode. While these methods are important in identifying modes and in directing control measures in human and agricultural diseases, quantifying the level of transmission by the different modes remains a challenge.

## 4. Genetic variation in transmission mode

The very diverse transmission modes that occur in closely related pathogen species suggests that the evolution of new transmission modes is ongoing and likely commonplace in nature. For example, many closely related strains of sexually transmitted diseases have both sexual and non-sexual transmission [12,56]. However, it is often not clear if transitions to a given transmission mode are simply the product of the host ecology and unrelated to genetic change. Quite drastic changes in transmission mode may not be contingent on any or only very little genetic change; the difficulty of distinguishing *Trypanosoma equiperdum* (causing dourine, a sexually transmitted disease in horses) from *T. brucei* (causing sleeping sickness transmitted by tsetse flies) suggests this host shift and transmission mode may have been possible with very little underlying genetic change [57]. Environmental differences favouring different parasite life cycle stages may also result in changes in transmission mode, and simply demonstrating differences among taxa may not necessarily reflect genetic changes [58,59].

Some of the best evidence we have for a genetic basis for transmission mode is the demonstration of specific genetic pathways leading to different tissue tropisms in closely related strains or species with contrasting transmission modes, e.g. genital and ocular chlamydia [12,60]. However, given the difficulty of quantifying transmission modes, it is perhaps not surprising that there appear to have been almost no studies on the quantitative genetics of transmission mode. Evidence of genetic control of transmission mode comes from the study of fungal endophytes that often act as partial ‘parasitic castrators’ producing fruiting bodies on the plant inflorescence (which produce horizontally transmitted spores), and whose hyphae invade the seeds, resulting in vertical transmission through the seed. Kover & Clay [61] showed fungal strains of *Atkinsonella* differed in the degree to which they induced the production of fruiting bodies, but vertical transmission was not studied. Tintjer *et al*. [62] showed that cloned genotypes of the grass *Elymus hystrix* infected with the fungus *Epichloë elymi* differed in the degree to which they produced fungal fruiting bodies responsible for horizontal transmission. However, all genotypes showed close to 100% vertical transmission of the fungus to the seeds, and thus there was no evidence of a trade-off with vertical transmission. These studies clearly show the importance of host factors in determining transmission mode (see also A. Brown 2016, personal communication).

Experimental studies have manipulated levels of horizontal and vertical transmission to study associated changes in pathogens. Stewart *et al*. [63] passaged barley stripe mosaic virus in barley, *Hordeum vulgare*, horizontally for four host generations and vertically for three generations. Each selection regime resulted in an increase in transmissibility by the respective mode, with clear trade-offs between them. In keeping with theoretical expectations, there was an increase in virulence by the horizontal mode and a decrease in virulence by the vertical mode, although levels of viral virulence did not reflect viral titer in the plants. Bull *et al*. [64] manipulated opportunities for vertical or horizontal transmission of bacteriophages infecting bacteria and found that when vertical transmission was promoted the viruses became less virulent. Similarly, Pagan *et al*. [65] selected for reduced pathogen virulence by serially passaging cucumber mosaic virus vertically in its host *Arabidopsis thaliana*, but no selection response was observed following horizontal transmission. Using the bacterium *Holospora undulata* infecting the protozoan *Paramecium caudatum*, Magalon *et al*. [66] demonstrated that populations of the host maintained below their carrying capacity selected for increased vertical transmission of the bacterium since high birth rates increased opportunities for vertical transmission. Dusi *et al*. [67] showed bacteria that had evolved in conditions promoting vertical transmission exhibited an almost complete loss of infectivity via the horizontal transmission route.

Phage λ viruses have a ‘genetic switch’ that, in one state, keeps them in a latent prophage state in the *Escherichia coli* genome such that they are vertically transmitted and resistant to superinfection. In the alternate state, usually turned on in response to stress, they initiate cell lysis and horizontal transmission. The sensitivity and threshold of this switch respond quickly to selection [68]. Spatial structure is expected to lead to selection for more ‘prudent’ (i.e. less virulent) pathogens, and correspondingly, Berngruber *et al*. [69], using competition between predominantly vertically and horizontally transmitting strains of phage *λ*, showed the latent state was favoured on an agar surface when the spatial structure was maintained, but not when it was disturbed, an outcome consistent with their theoretical expectations. A thorough knowledge of the genetic basis for alternative transmission modes makes phage *λ* a very useful system for experimental studies.

However, the outcome of selection experiments is also not always as expected. Turner *et al*. [70] allowed plasmids to evolve for 500 generations in populations of bacteria that differed in density, and found no evidence of response to selection for vertical or horizontal transmission.

## 5. Trade-offs and transmission modes

While it would be obviously advantageous for a pathogen to use all possible transmission routes, as in any evolutionary process involving a complex phenotype, there are likely to be direct trade-offs between these routes themselves or these routes may have other indirect fitness effects. In an evolutionary context, trade-offs are quantified by measuring the genetic correlations between different traits: a negative genetic correlation between alternative transmission modes suggests that increasing one transmission mode would decrease the other. However, we know of no data on estimates of genetic correlations between transmission mode and other fitness components, in either pathogens or hosts.

It has been commonplace in theoretical and general discussion to expect trade-offs in transmission mode. This is most obvious in the conflict between vertical and horizontal transmission. Activities of a host or parasite that increase the rate of horizontal transmission (e.g. greater production of infectious particles) may increase mortality or decrease reproduction, and this will correspondingly reduce vertical transmission of the parasite via the offspring, necessarily leading to an evolutionary trade-off [7,64,71,72]. Correspondingly, theory predicts that there should be a trade-off between pathogen virulence and transmission mode [73]. If the pathogen kills the host quickly there is a cost in terms of a reduced number of infectious particles, which decreases horizontal transmission. At low host densities, contact rates between host and pathogen may drop below the threshold necessary for persistence [74], so that persistence is more likely if the pathogen can be vertically transmitted and has a low virulence so the host survives till reproduction.

These concepts seem intuitive when considering, for example, the insect baculoviruses, which are invariably lethal when horizontally transmitted but are largely asymptomatic when vertically transmitted [75]. Natural populations of insects are often characterized by large seasonal variation in abundance, including a complete absence of stages that transmit horizontally; hence, such populations harbour covert baculovirus infections that are vertically transmitted [76]. Another example is the protozoan parasite *Ophryocystis elektroscirrha* of monarch butterflies, *Danaus plexippus,* which is transmitted horizontally when adult butterflies ingest spores on host plant leaves, and vertically when spores are transmitted on the outside of the eggs [77]. As expected, strains of the parasite that produce more spores are transmitted more effectively horizontally (to other adults via the leaves). However, their vertical transmission is actually reduced because larger numbers of spores on the eggs cause more severe infections that lead to premature death of the larvae and pupae. However, these strains are efficiently horizontally transmitted because they leave more spores on the leaves. Similar trade-offs are seen in a wide range of host–pathogen systems, from malaria [78] to microsporidia [79], myxozoans [80] and bacteriophage [64].

The shape of the trade-off is likely to be important in determining whether evolutionary changes lead predominantly to one mixed mode, or to the maintenance of both modes as genetic variants with alternative pathways [81]. This is because the trade-off shape is critical in determining the outcome of evolutionary predictions. The measurement of the shape of the trade-off also presents particular challenges, because estimates of genetic correlations *per se* cannot incorporate nonlinearities (other than by transformation) and so we lack the statistical tools for estimating nonlinear genetic trade-offs. The shape of the trade-off curve is also critical in determining the outcomes of coevolution between hosts and pathogens with regard to resistance and infectivity [82,83].

The dependency of trade-offs on environmental conditions also needs to be considered [84]. Intriguingly, research on microsporidians in mosquitoes has shown that the factors influencing selection on vertical versus horizontal transmission include food availability and whether the parasites are embedded in co-infections [85]. Long-term environmental changes in SO_2_ levels, by affecting the likelihood of infection via leaves, has been posited as the cause of shifts between leaf-to-leaf (horizontal) and seed (vertical) transmission of the fungal pathogen of wheat, *Phaeosphaeria nodorum* [86].

## 6. Evolutionary pathways in transmission mode

### (a) Population genetics theory

While there have been many studies positing the advantages or otherwise of different transmission modes, some studies have addressed the evolution of transmission mode specifically from a population genetics standpoint, asking how allele frequencies determining transmission mode are likely to change, and with what outcome. Thrall & Antonovics [56], observing that sexually transmitted diseases (such as chlamydia, syphilis, HSV-2 and pubic lice) often have non-sexually transmitted counterparts (strains or closely related species), asked whether it was possible to maintain genetic polymorphisms in transmission mode even when the strains excluded each other (directly or immunologically) from a single host. They implicitly assumed a complete trade-off in transmission mode, such that each genotype could transmit either sexually (in a frequency-dependent manner) or non-sexually (in a density-dependent manner), and showed that stable genetic polymorphism in alternative transmission modes was possible. This was even when the pathogen strains were excluding each other on the same host resource, illustrating how ‘Gause's Principle’ (that two species using the same resource cannot coexist) could be violated by the complexities of transmission.

There have also been applications of adaptive dynamics theory to transmission mode evolution. In a thorough analysis of the evolution of vertical versus horizontal transmission, Ferdy & Godelle [81] examined the consequences of different forms of the trade-off between vertical and horizontal transmission. They also showed that polymorphism in transmission mode was possible if the trade-off was convex (e.g. increased horizontal transmission, if it causes sterility, will not continue to decrease vertical transmission proportionately); but if the trade-off was concave, then mixed-mode transmission of one genotype was favoured (e.g. in a situation where increased horizontal transmission that increases mortality continues to decrease vertical transmission). Their model included competition among the symbionts for resources within the host, and this complicates the outcomes, depending on the interaction within the host.

A strong theoretical framework for the study of transmission mode, especially the evolution of vector transmission, was developed by Gandon [87] in the context of epidemiological and genetic dynamics of two (and multi) host systems. Using this framework, Gandon identified the forces leading to a second host acting as an effective vector, and showed that there was a positive feedback between evolution of vector transmission and evolution of virulence, as postulated by Ewald [4] many years previously. Using this framework, he also showed that different transmission routes (i.e. involving different hosts) could result in evolutionary branching and polymorphism, depending on the form of the trade-offs between virulence, pathogen multiplication and host susceptibilities.

The evolution of transmission mode in relation to virulence is important from an applied perspective. Thus, if highly virulent strains can coexist with non-virulent ones, very serious health consequences of disease in a subset of the population may be due to virulent pathogen variants. This may be less desirable than the presence of only one strain of intermediate virulence. Boldin & Kisdi [88] investigated this in diseases that had both environmental and direct host-to-host transmission, the worry being that environmentally transmitted genotypes might show higher virulence, as their persistence would be less compromised by a shortened host lifespan. Here too, stable genetic polymorphisms could be maintained; however, the polymorphism generally involved strains less virulent than would be expected under one transmission mode or the other. van den Bosch *et al*. [86] used a similar approach to investigate levels of vertical (seed) versus horizontal (leaf-to-leaf) transmission in a fungal disease (*Phaeosphaeria*) of wheat. They showed evolutionary ‘bi-stability’ in pathogen ‘aggressiveness’ (i.e. disease severity or virulence) and therefore the potential for polymorphisms in degree of vertical (seed) and horizontal (leaf) transmission mode under a wide range of conditions.

Several points stand out from these theoretical studies. The first is that, relative to the evolution of virulence, the evolution of transmission mode has received less attention from population geneticists, even though the results can often be illuminating theoretically and of applied significance in understanding virulence. Polymorphisms in transmission mode are possible, and defining the circumstances under which polymorphic genotypes versus multiple transmission modes in one genotype are favoured remains a challenge. This stands in contrast with our understanding of the evolution of host–pathogen interactions in infectivity and resistance [1]. Additionally, it should be noted that most studies have assumed that transmission is under ‘pathogen control’, i.e. that it is genetic variation in the pathogen rather than in the host that is driving the evolution of transmission mode, even though the frameworks for doing otherwise are well established in theory [87,88]. It remains to be seen whether more complex ‘transmission-genetics’ makes other coevolutionary scenarios possible, in a way analogous to what is seen with genetics of resistance and infectivity.

### (b) Examples of evolutionary changes in transmission mode

The general perceived ‘adaptationist’ wisdom is that transmission mode will evolve in the direction of where there is the greatest transmission opportunity at least cost (i.e. the mode and route that produce the greatest fitness gains for the pathogen). For example, it has been argued that decreasing host density, or periods of low density, will favour vertical [10] or sexual transmission [89], while high density will favour aerial or (non-sexual) direct contact transmission. However, there will also be selection on hosts to decrease transmission, and the force of this selection will differ among transmission modes. For example, in primates, several immunological parameters appear to be determined largely by the degree of sexual transmission rather than by other transmission modes [90]. Moreover, if there are two potential pathways, such as ocular or genital transmission, it may be easier/less costly for the host to evolve resistance via one route rather than another. Age specificity of resistance may also determine whether a disease is transmitted aerially to offspring or sexually via reproduction among adults [91].

In the following sections, we review a selection of phylogenetic studies that address how evolutionary changes in transmission mode may have occurred in the past. Most of them have focused on pathogens as the anticipated driver of transmission mode.

#### (i) Vertical versus horizontal transmission

Sachs *et al*. [92] reviewed the evolutionary transitions within bacterial symbionts, focusing mostly on mutualistic relationships. They concluded that free-living forms preceded host-associated ones and that horizontal transmission was the most basal type and occurred when bacteria were acquired from the outside environment. Exclusive vertical transmission was rare (of 127 host-associated bacteria, 108 were horizontally transmitted, 14 vertically transmitted and five had mixed-mode transmission). Of the vertically transmitted species, three were considered to be parasitic, 11 mutualistic. Sachs *et al*. [92] suggested vertical transmission is often an evolutionary end point that is irreversible because of the negative genetic effects (accumulation of mutations and gene loss) that strict vertical transmission may have on the symbiont. Moran *et al*. [10], focusing on heritable (vertically transmitted) insect endosymbionts, showed that obligate (vertically transmitted) and facultative (horizontally transmitted) symbionts have evolved several times. In *Rickettsia,* Perlman *et al*. [93] showed that while most species are vertically transmitted symbionts of invertebrates, some have later become horizontally (by invertebrate vectors) transmitted pathogens of vertebrates. The comparison between *Coxiella burnetii* and *Coxiella*-like endosymbionts of ticks is also relevant. *Coxiella*-like bacteria are maternally inherited and potentially mutualistic bacteria in ticks. *Coxiella burnetii* causes Q-fever in humans and infects a variety of vertebrate species and is transmitted horizontally through many different routes. Recent studies have shown that *C. burnetii* recently evolved from an inherited symbiont of ticks that succeeded in infecting vertebrates [94].

While horizontal transmission of Microsporidia is the most common mode of transmission, phylogenetic data show that vertical transmission has evolved several times in diverse lineages [87]. Vertical transmission might be under-reported because of the low virulence of vertically transmitted parasites [95].

Brown & Akçay examined whether transmission modes in a range of grass/epichloe interactions are correlated with host or symbiont evolutionary history. They found that signals of host evolutionary determination of transmission were present, but they depended on the particular symbiont. However, there was no phylogenetic signal in the symbiont effect. They interpreted this as suggesting that faster evolution in the symbiont masked any phylogenetic signal, whereas in the host this signal was more conserved. The joint phylogenetic analysis of host and symbiont traits is an important future direction as disease traits are a likely to be a consequence of the evolutionary history of both the host and symbiont.

#### (ii) Sexual versus non-sexual transmission

There are arguments for expecting sexual transmission to be ancestral to non-sexual transmission. Frequency-dependent transmission allows the persistence of pathogens at low population densities, and therefore protects against bottleneck events. Sexually transmitted diseases are often persistent in the host, and this increases their likelihood of being carried by a host migrating to a new location, as are covert infections [96]. Because sexual reproduction is a regular feature of the life cycle, sexual transmission may be considered relatively ‘reliable’. On the other hand, sexual transmission severely limits opportunities for cross-species transmission. Sexually transmitted pathogens have lower host ranges [7], which might be a critical factor in determining long-term persistence on alternative hosts. Antonovics *et al*. [12] explored whether sexual transmission was ancestral or derived by mapping transmission mode onto phylogenies of pathogens. The results showed that it seemed more common for sexual transmission to be a derived trait rather than ancestral, and also that sexual transmission appeared to have evolved in an extremely diverse way, and often repeatedly as in the *Chlamydias* and human papilloma viruses. However, determination of the evolutionary pathways was very difficult, less because of a lack of reliable phylogenies and more because of accurate/reliable information on transmission mode.

#### (iii) Evolution of complex life cycles in helminths

Complex life cycles, where several life stages of a parasite are found in different hosts, are a remarkable feature of both animal and plant parasites. The hosts in such life cycles can be extremely unrelated phylogenetically, making it hard to envisage how such ‘host shifts’ could ever have occurred. Moreover, the occurrence of a parasite on phylogenetically distinct hosts raises the question of whether the evolutionarily more ancestral host represents the ‘original’ host; alternatively, it can be posited that the original host is the ‘definitive’ host (i.e. in which sexual reproduction occurs) and that the non-definitive host has been acquired subsequently. For example, did digenean trematodes, which alternate between sexual stages in the vertebrate host and asexual stages in snails, evolve parasitism in vertebrates and then acquire the snail hosts, or were they originally parasites of molluscs? The phylogenetic evidence on this specific point is somewhat ambiguous because the common ancestor of the digeneans and all the Neodermata is inferred to have had both the vertebrate and invertebrate host [97,98]. However, tracing the phylogeny even further back and placing it the context of the fossil record is problematic because of limited taxon sampling; an invertebrate host is, therefore, often inferred based on the expectation that such hosts should be ancestral to vertebrates [99].

Many authors have speculated on the pathways whereby parasites could gain new hosts and establish complex life cycles. Much of the focus has been on the helminths (flatworms, tapeworms and nematodes) where this pattern is very prevalent [100–102]. For instance, parasites of the original host species may evolve to exploit that species’ predators, a process that has been termed ‘upward incorporation’. Such incorporation might be driven by increased parasite fecundity in larger predator hosts. For example, upward incorporation appears to have occurred when ancestral acanthocephalans, endoparasites of marine arthropods, incorporated a vertebrate predator as a second host [103,104]. Upward incorporation to a new definitive host may also increase parasite densities, and lead to an increased probability of finding a sexual partner [105,106] or to a decrease in inbreeding because of multiple infections of a larger host [107]. In digenean trematodes, acquisition of a second intermediate (paratenic) host may also enable an increased intermixture of genotypes from the snail host within which the parasites multiply only asexually. The difficulty of accounting for such life cycles has also led to some extreme hypotheses. For example, Smith Trail [108] proposed that infected hosts might benefit by ‘submitting to’ predation if suicide is repaid by inclusive fitness gains when close relatives experience reduced infection. Subsequently, parasite survival in the host's predator generated a complex life cycle by upward incorporation.

Alternatively, when the new host is at a lower trophic level, there may have been ‘downward incorporation’ [101]. Prey of the original host may frequently have ingested parasite transmission stages because of their proximity to the original host and thereby may have become intermediate hosts. Being prey to the original host may enhance transmission back to that host [106]. Such downward incorporation has been associated with the occurrence of a ‘trophic vacuum’, i.e. the difficulty of transmission of small free-living infective stages among hosts at a higher trophic level where the animals are large and at low density [109]. Platyhelminthes appear to present such an example of downward incorporation: the lineage ancestral to digeneans and cestodes has become parasitic in invertebrates [101]. Paratenic hosts may also be acquired by downward incorporation as a means of increasing transmission [106]. Intermediate hosts could also be added via ‘lateral incorporation’ if the parasite has multiple hosts involved; in a generalist pathogen each of two parasite stages come to specialize on one of the hosts [106].

It would be exciting to incorporate many of these verbal arguments from evolutionary ecology into a more rigorous genetic and ecological framework, as this may lead to a broader range of testable predictions [88].

#### (iv) The evolution of transmission by arthropod vectors

Blood-feeding arthropods such as mosquitoes and ticks transmit a broad range of microorganisms that cause disease in vertebrates. Some vector-borne pathogens can also be transmitted via other modes such as direct contact, vertical transmission or aerosol transmission, in many cases at a low rate (for example, dengue virus [110]). How might such a system evolve? Possible precursors to vector-borne parasites could have been exclusively arthropod pathogens that infected a dead-end vertebrate host and acquired the ability to cause transmissible infections; this would be equivalent to ‘downward’ incorporation in the context of helminths. An intermediate step here could be non-systemic transmission during co-feeding, in which a pathogen could spread between co-feeding haematophagous arthropods via a feeding site on a host without the host necessarily becoming infected [111].

Alternatively, an exclusively vertebrate pathogen that is repeatedly ingested by an arthropod proto-vector during blood-feeding could acquire the ability to infect it; there is a parallel here with ‘upward incorporation’. An intermediate step here could be mechanical transmission, in which a pathogen is transmitted by a blood-feeding insect without any fitness cost as no replication occurs in the insect. Mechanical transmission is seen in a broad range of vector-borne pathogens. Some vector-borne pathogens have also lost the ability to be biologically transmitted altogether; thus *Trypanosoma evansi* has lost the ability to replicate in insects even though they remain important vectors [112]. The third possibility is that a pathogen may already be infecting both vertebrate and invertebrate hosts and is initially transmitted within and between them via alternative transmission routes, but these may then become restricted to cross-species only transmissions.

Phylogenetic analyses of arthropod-borne viruses (arboviruses) provide several examples of viral groups where it appears that the ancestral virus initially infected arthropods (insects, in the case of flaviviruses [113]; ticks, in the case of orbiviruses [114]), but later acquired vertebrate hosts. Subsequently, these have become transmissible by yet other blood-feeding arthropod groups. Reversals of this process can also occur; a study of the host associations of rhabdoviruses vectored by arthropods showed that arthropod-specific viruses had arisen, albeit rarely [115]. The evolutionary origin of another main group of arboviruses, the alphaviruses, remains unknown as they are all known or suspected to be arthropod-borne [116].

The flaviviruses and orbiviruses most strongly support the scenarios of the arthropod host being ancestral, although in the case of insectivorous vertebrate hosts it could also plausibly be explained by vertebrate hosts becoming orally infected by ingesting infected arthropods [117,118]. A similar evolutionary history has been reported for *C. burnetii*, the causative agent of Q-fever [96].

## 7. Host shifts and changes in transmission mode

A large number of emerging infectious diseases are the result of parasite shifts from one host species to another [119,120]. Different modes of transmission may occur in novel host species due to host genetic, social and ecological factors affecting the epidemiological spread of the pathogen.

Understanding how transmission evolves following host shifts is of major importance when considering the emergence of infectious disease in humans. For example, in aquatic birds influenza A viruses appear to be largely spread environmentally via the faecal–oral route [121]. However, in mammals, influenza viruses must evolve aerial transmission to spread successfully between individuals [122]. These shifts in transmission are due to differences in host receptor binding, with avian influenza having to adapt in mammalian hosts to different sialic acid receptors with different tissue distributions [123].

HIV-1, which is largely responsible for the AIDS pandemic in humans, is the result of host shifts of viruses from chimpanzees and gorillas into humans [124]. How simian immunodeficiency viruses (SIV, the non-human primate forms of HIV) are transmitted in natural populations of primates is poorly understood. A study examining SIV transmission in semi-natural mandrill populations found that transmission is correlated with maternal kinship yet is not transmitted maternally, suggesting that behavioural interactions between related juveniles facilitate transmission [125]. This differs from HIV in humans where transmission is largely sexual and vertical (maternal), or through infected blood. Surprisingly, even though HIV phylogeny is well understood, functional studies have not examined whether the change in transmission mode is due to evolutionary changes in the pathogen, or if there are simply different transmission opportunities in different host species.

Endophytic fungi from the genus *Epichloë* show evidence of divergence in transmission mode following host shifts. Different lineages of the fungi appear to have emerged through host shifts between grass species, with associated changes in reproduction and transmission mode. Some species reproduce sexually and are horizontally transmitted and others reproduce asexually and are vertically transmitted [126].

The maternally transmitted endosymbiont *Wolbachia* uses various forms of reproductive manipulation to maximize its transmission and ensure its persistence in host populations [127]. However, it has been shown experimentally that *Wolbachia* can change phenotype directly following a host shift. For example, a *Wolbachia* strain that causes cytoplasmic incompatibility in *Drosophila recens* causes males to die in a new host, *D. subquinaria* [128]. A similar change has been observed in a host shift of *Wolbachia* between two species of Lepidoptera [129], and the inverse pattern in shifts of male killing strains when they are moved into different *Drosophila* species [130]. These changes in phenotype seem to be due to host factors and the expression of existing genotypes rather than *de novo* evolution of the pathogens/symbionts. This suggests that these bacteria maintain the genetic capability to express multiple modes of transmission. A study of five virus families found that viral speciation events were primarily associated with host shifts rather than with changes in tissue tropism within the host [131]. Similar tissue tropisms suggest similar routes of transmission rather than changes in transmission mode by the pathogen.

## 8. Evolution of transmission mode and human disease

Changes in transmission mode are often involved in disease emergence, and it remains a matter of urgency to determine with confidence whether new transmission modes may evolve in extant disease threats or if currently minor transmission modes could become major routes given new circumstances and opportunities. Thus, in the recent Ebola epidemic, there were fears that the Ebola virus might evolve aerial transmission given greater opportunities for this mode of transmission in crowded human situations [132], especially as aerosol transmission of filoviruses has been shown in laboratory experiments [133,134]. Similarly, the possibility of sexual routes of infection of not only Ebola, but also Zika virus [135] beg the serious question of whether such routes might become more important because of evolutionary changes under new transmission opportunities. Explicit consideration of ‘why’ particular routes of transmission do or do not evolve has been rare. Day *et al*. [136] discussed why HIV appeared not to have evolved vector transmission (via blood meals) and, among other possibilities, argued that this was because such transmission might have been quickly lethal and therefore the pathogen would have had a low fitness. Unfortunately, we simply do not have enough knowledge of the kinds of mutational steps that would be needed for changes in transmission mode to happen, whether such changes would have associated costs, nor of the circumstances that would favour their spread. There is clearly some urgency in addressing such issues in a rigorous way at a functional, comparative and experimental level.

Transmission mode has strong evolutionary consequences for disease severity, and conversely changes in disease severity due to treatment could result in evolutionary changes in transmission mode, in an analogous way to concerns that vaccination policies may change pathogen replication rate and therefore virulence. There is substantial circumstantial evidence that historical changes towards reduced virulence of syphilis were associated with a shift from non-sexual to sexual transmission [137].

## 9. Conclusion

The evolution of transmission mode presents a fascinating medley of challenges for the future, ranging from theoretical exploration of transmission in a coevolutionary setting, to explaining startling biological conundrums such as the evolution of complex life cycles. It is very clear that there are many different ideas and approaches, but it is a difficult field where even simply quantifying the phenotype, i.e. the contributions of different transmission modes and routes to pathogen and host fitness, is a huge hurdle. In the context of human diseases, there is a remarkable lack of understanding ‘why’ and ‘when’ different transmission modes are likely to evolve, and whether changed circumstances following pathogen entry into a human population would result in the evolutionary amplification of a particular transmission pathway. This applied imperative is sufficient reason to see research into the evolution of transmission as an important continuing endeavour.
